# Destabilization of light NREM sleep by thalamic PLCβ4 deletion impairs sleep-dependent memory consolidation

**DOI:** 10.1038/s41598-020-64377-7

**Published:** 2020-06-01

**Authors:** Joohyeon Hong, Go Eun Ha, Hankyul Kwak, Yelin Lee, Hyeonyeong Jeong, Pann-Ghill Suh, Eunji Cheong

**Affiliations:** 1 Department of Biotechnology, College of Life Science and Biotechnology, Yonsei University, Seoul, 03722 Republic of Korea; 2 School of Life Science, Ulsan National Institute of Science and Technology, Ulsan, 44919 Republic of Korea; 3 Korea Brain Research Institute, Daegu, 41062 Republic of Korea

**Keywords:** Non-REM sleep, Long-term memory

## Abstract

Sleep abnormality often accompanies the impairment of cognitive function. Both rapid eye movement (REM) and non-REM (NREM) sleep have associated with improved memory performance. However, the role of composition in NREM sleep, consisting of light and deep NREM, for memory formation is not fully understood. We investigated how the dynamics of NREM sleep states influence memory consolidation. Thalamocortical (TC) neuron-specific phospholipase C β4 (PLCβ4) knockout (KO) increased the total duration of NREM sleep, consisting of destabilized light NREM and stabilized deep NREM. Surprisingly, the longer NREM sleep did not improve memory consolidation but rather impaired it in TC-specific PLCβ4 KO mice. Memory function was positively correlated with the stability of light NREM and spindle activity occurring in maintained light NREM period. Our study suggests that a single molecule, PLCβ4, in TC neurons is critical for tuning the NREM sleep states and thus affects sleep-dependent memory formation.

## Introduction

Sleep is a fundamental biological process that is well-preserved across most animals^1^. Sleep has crucial roles in brain metabolite clearance^2^, energy conservation^3^, brain plasticity^4^, and the process of cognition or emotions^5^. Many human studies suggest that the quantity and quality of sleep, such as duration, latency, architecture, and brain rhythms, affect memory function^6–12^. They report that a longer sleep duration supports memory consolidation^7^, whereas sleep disruption is accompanied by memory deficits^8,9^. Impaired memory consolidation has been associated with shorter sleep duration and altered sleep-brain rhythms in several psychiatric disorders, such as major depression and schizophrenia^10,11^. Moreover, memory performance in psychiatric patients can be improved after sleep^12^. Thus, sleep is thought to benefit memory consolidation.

Sleep consists of non-rapid eye movement (NREM) and rapid eye movement (REM) sleep. In humans, NREM sleep is further subdivided into stages 1–4 according to sleep depth^13^. NREM stages 1 and 2 (N1 and N2) are classified as light NREM sleep. The N2 stage is characterized by the occurrence of a K-complex (<1 Hz) and a sleep spindle (12–16 Hz in humans and 10–15 Hz in rodents) in electroencephalography (EEG) activity. N3 and N4 stages are classified as deep NREM sleep and are characterized by large-amplitude, low-frequency delta (δ) waves (0.5–4 Hz) in EEG activity. In most studies using rodent models, NREM sleep is not categorized into N1–N4 stages. REM sleep is marked by distinctive regular theta (θ) waves. Several studies have investigated the effects of NREM versus REM sleep on memory formation to elucidate whether different sleep stages have distinct roles in various types of memories^14,15^. Human studies have suggested that deep NREM sleep predominantly benefits declarative memory consolidation (e.g., remembering events or facts), whereas REM sleep supports the consolidation of non-declarative memories (e.g., learning languages or movement-based skills)^15,16^. However, the role of light NREM sleep on memory consolidation is relatively unknown^17^.

During NREM sleep, large-amplitude oscillatory brain rhythms reflect the ensemble activities in thalamocortical circuit, which is composed of cortical, thalamocortical (TC) and thalamic reticular nuclei^18^. The thalamocortical circuit is thought to be the origin of sleep spindles and δ waves, which primarily occur in light and deep NREM sleep, respectively^19,20^. Among the many inputs to TC neurons, the corticothalamic pathway from cortical neurons in layer VI sends massive glutamatergic inputs^21^. The activation of corticothalamic inputs has been reported to increase the excitability of TC neurons via the type 1 metabotropic glutamate receptor (mGluR1)^22^. mGluR1s are exclusively found in postsynaptic membranes that receive corticothalamic inputs, and are tightly linked to phospholipase C (PLC) β4 in TC neurons^23,24^. Several studies report that PLCβ4 is highly expressed in TC neurons and is involved in regulating the firing properties of TC neurons, which affect thalamocortical oscillations^25,26^. In the present study, we investigated changes in light and deep NREM sleep and their effects on memory consolidation using TC-specific PLCβ4 knockout (KO) mice.

We found that TC-specific deletion of PLCβ4 impaired sleep-dependent declarative memory consolidation, although the total duration of NREM sleep increased. Memory function was positively correlated with light NREM stability and spindle activity. The current results suggest that the thalamocortical circuit affects the dynamics of light and deep NREM sleep. Furthermore, light NREM sleep, and not just the total duration of NREM sleep, seem to be important for memory formation.

## Results

### TC-specific PLCβ4 KO decreased the duration of light NREM sleep

**Figure 1:**
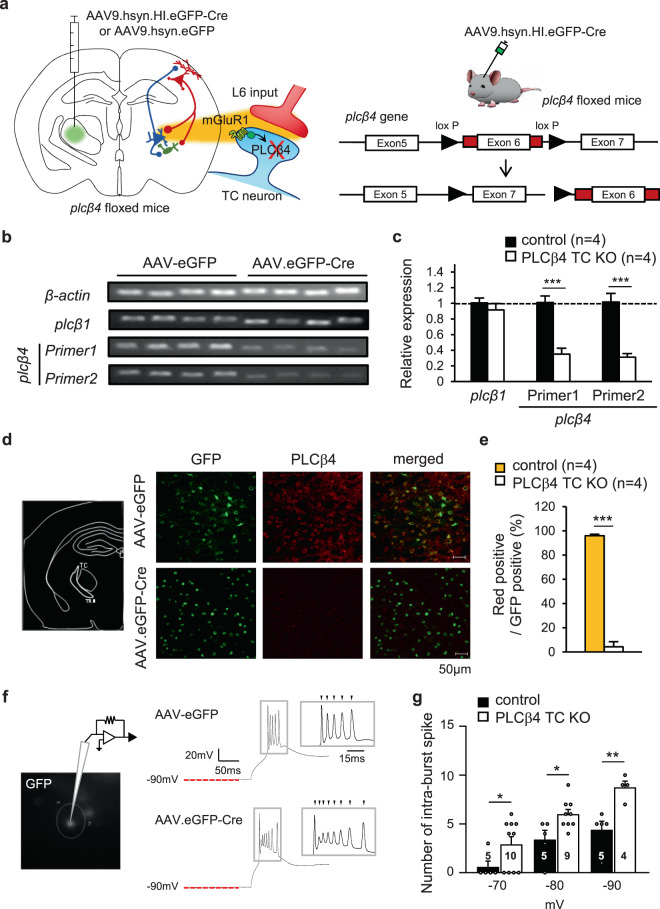
The deletion of PLCβ4 in thalamic neurons of *plcβ4* floxed mice. (**a**) Schematic showing bilateral injection of AAV9.hsyn.HI.eGFP-Cre.WPRE. SV40 (PLCβ4 TC KO) or AAV9. hsyn.eGFP.WPRE.Bgh (control) in the TC regions of *Plcβ4* floxed transgenic mice. Exons 6 in alleles of the *Plcβ4* gene was deleted by Cre recombinase (right panel). **(b**) RT-PCR data assessing *Plcβ4* expression for confirmation of the Cre-loxP system. The grouping of gels were cropped from different gels by genes (*Plcβ1, Plcβ4* (Primer 1,2) and *β-*actin). Uncropped gels are shown in Supplementary Fig. S1. **(c)** The quantitative mRNA expression levels of *Plcβ1* and *Plcβ4* normalized to *β-actin* (control, n = 4, black bar; PLCβ4 TC KO, n = 4, white bar). **(d)** Representative fluorescence image of viral expression (green) in the TC regions with PLCβ4 staining (red). Scale bar represents 50 µm. **(e)** The quantitative PLCβ4 expression was reduced in TC neurons of PLCβ4 TC KO mice (control, n = 4, yellow bar; PLCβ4 TC KO, n = 4, white bar). **(f)** Representative traces of rebound burst firing recorded from TC neurons in ventrobasal complex region infected with AAV-eGFP or AAV-eGFP-Cre under current-clamp configuration. **(g)** The number of intra-burst spikes at −70, −80, or −90 mV. (control, n = 5, black bar; PLCβ4 TC KO, −70 mV; n = 10, −80 mV; n = 9, −90 mV; n = 4, white bar). Data are represented as the mean ± standard error of the mean (SEM). ^*^p < 0.05; ^**^p < 0.01; ^***^p < 0.005.

To examine the effect of the TC-specific PLCβ4 deletion on the natural sleep-wake pattern, AAV.eGFP (control) or AAV.eGFP-Cre (PLCβ4 TC KO) were injected into the TC region of *Plcβ4* floxed mice (Fig. 1a). The mRNA expression levels of thalamic *Plcβ4* were significantly lower in PLCβ4 TC KO mice than in control mice (Primer 1: control, 1.01 ± 0.08; PLCβ4 TC KO, 0.35 ± 0.07; p = 0.001, unpaired t-tests; Primer 2: control, 1.02 ± 0.11; PLCβ4 TC KO, 0.31 ± 0.05; p = 0.004, unpaired t-tests; Fig. 1b,c). Additionally, we confirmed that AAV.eGFP was expressed in the cytosol and nuclei of TC neuronal cells (Fig. 1d). AAV.eGFP-Cre was specifically translocated to the nuclei of TC neurons and caused a significant reduction of PLCβ4 protein expression (AAV-eGFP, 95.96 ± 1.34%; AAV.eGFP-Cre, 4.26 ± 4.26%; p = 7×10^−5^, unpaired t-tests; Fig. 1d,e). We further investigated the firing pattern of TC neurons labeled with GFP expression in control and PLCβ4 TC KO mice (Fig. 1f) and found that the number of spike per burst was significantly increased in PLCβ4 KO TC neurons compared with control following various hyperpolarizing steps (−70mV: control, 0.6 ± 0.6; PLCβ4 TC KO, 2.9 ± 0.8; p = 0.04, unpaired t-tests; −80mV: control, 3.4 ± 0.9; PLCβ4 TC KO, 6.0 ± 0.5; p = 0.046, unpaired t-tests; -90mV: control, 4.4 ± 0.9; PLCβ4 TC KO, 8.8 ± 0.6; p = 0.007, unpaired t-tests; Fig. 1g).

**Figure 2:**
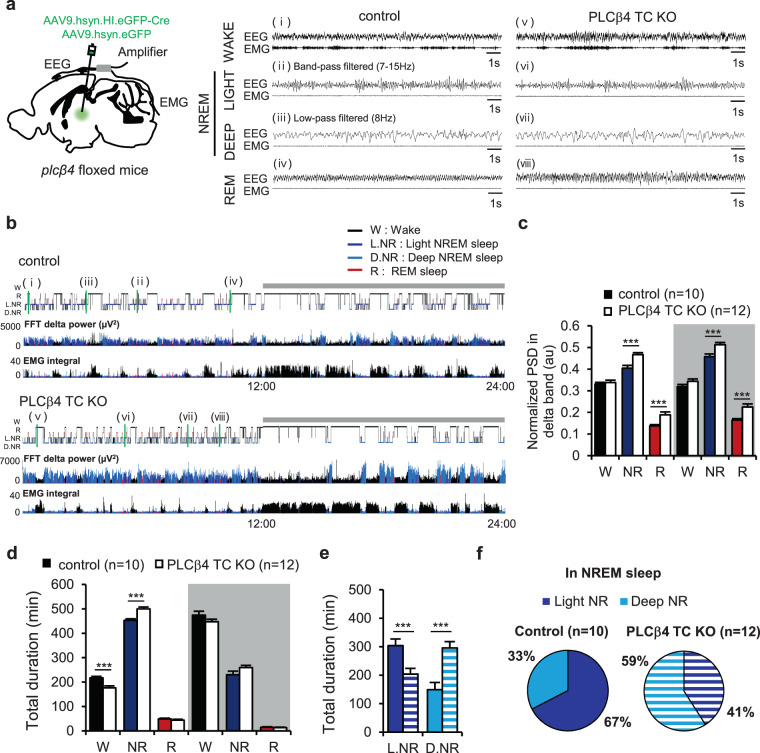
The reversed composition of light and deep NREM sleep by TC-specific PLCβ4 deletion. (**a**) Representative EEG and EMG traces during awake, light NREM, deep NREM, and REM sleep. The position of each trace (i–viii) was indicated in the hypnogram. **(b)** Representative hypnograms with fast Fourier transformation-derived δ band power and integrated EMG activity over 24 h. The y-axis of the hypnograms shows the state of vigilance, and the x-axis shows the 24 h period that included one light and one dark (gray bars) cycle. **(c)** Normalized power spectrum density (PSD) for the δ bands (arbitrary units, au) in awake (W), NREM (NR), and REM (R) sleep during the light and dark phases. **(d)** The total time spent in W, NR, and R sleep during the light and dark phases. **(e)** The total time spent in the light NREM (L.NR) and deep NREM (D.NR) sleep during the light phase. **(f)** The average percentage of L.NR and D.NR in total NREM sleep in the control and PLCβ4 TC KO mice (control, n = 10, colored bar; PLCβ4 TC KO, n = 12, white or graduated bar). Data are represented as the mean ± standard error of the mean (SEM). ^***^p < 0.005.

Both the control and PLCβ4 TC KO mice displayed typical and characteristic EEG and electromyography (EMG) patterns of wake and sleep states (Fig. 2a). The transition from wake to NREM sleep showed a reduction of EMG tone with high amplitudes and slow EEG patterns. NREM sleep was further subdivided into light (L.NR, ii and vi) and deep (D.NR, iii and vii) NREM states based on the ratio of δ band power^27–30^. Typically, low amplitude, regular EEG patterns in the θ frequency range were observed in REM sleep with EMG atonia (iv and viii). The hypnogram showing the change of vigilance state over 24 h revealed normal nocturnal activity with a diurnal sleep preference in both groups. It is noteworthy that NREM sleep mostly comprised light NREM in control mice, but mostly deep NREM in PLCβ4 TC KO mice (Fig. 2b). In brain rhythms, the δ band power was enhanced in PLCβ4 TC KO mice compared with control mice in NREM and REM sleep state during the light and dark phase (Light phase; NREM: control, 0.40 ± 0.01; PLCβ4 TC KO, 0.47 ± 0.009; p = 0.0007, unpaired t-tests; REM: control, 0.14 ± 0.005; PLCβ4 TC KO, 0.19 ± 0.01; p = 0.0003, unpaired t-tests; Dark phase; NREM: control, 0.46 ± 0.01; PLCβ4 TC KO, 0.51 ± 0.01; p = 0.0003, unpaired t-tests; REM: control, 0.16 ± 0.006; PLCβ4 TC KO, 0.22 ± 0.014; p = 0.0015, unpaired t-tests; Fig. 2c).

The total duration of NREM sleep significantly increased with a reduction of wake amount in PLCβ4 TC KO mice compared with control mice during the light phase (Wake: control, 217.63 ± 5.96 min; PLCβ4 TC KO, 176.34 ± 7.85 min; p = 0.0005, unpaired t-tests; NREM: control, 453.01 ± 6.37 min; PLCβ4 TC KO, 499.844 ± 7.91 min; p = 0.0002, unpaired t-tests; Fig. 2d). However, the total duration of REM sleep was unchanged in PLCβ4 TC KO mice compared with control mice (Fig. 2d). In the dark phase, there were no differences in the total duration of wake and sleep states between the two groups (Fig. 2d). In NREM sleep, the total duration of light NREM significantly decreased, whereas the duration of deep NREM was elevated in PLCβ4 TC KO mice compared with control mice during the light phase (L.NR; control, 304.03 ± 23.12 min; PLCβ4 TC KO, 203.92 ± 20.04 min; p = 0.004, unpaired t-tests; D.NR; control, 148.99 ± 25.56 min; PLCβ4 TC KO, 295.92 ± 21.80 min; p = 0.0003, unpaired t-tests; Fig. 2e). It is noteworthy that NREM sleep consisted of a greater percentage of light NREM than the deep NREM in control mice, but this pattern was reversed in PLCβ4 TC KO mice, which exhibited much longer deep NREM sleep (L.NR; control, 67.41 ± 5.42%; PLCβ4 TC KO, 40.90 ± 4.13%; p = 0.001, unpaired t-tests; D.NR; control, 32.59 ± 5.42%; PLCβ4 TC KO, 59.10 ± 4.13%; p = 0.001, unpaired t-tests; Fig. 2f). These results suggest that TC-specific PLCβ4 deletion decreased the duration of light NREM sleep, yet increased the total duration of NREM sleep.

### **Each episode of light NREM sleep was destabilized whereas episodes of deep** NREM **sleep were more frequent and longer in TC-specific PLCβ4 KO mice**

**Figure 3:**
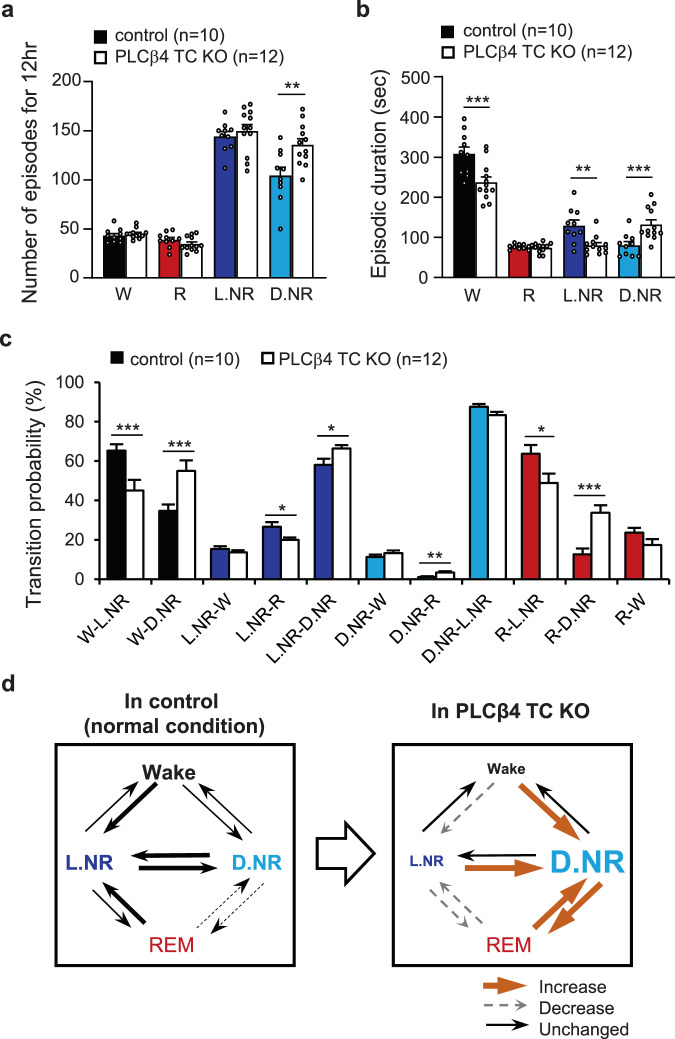
Altered dynamics in light and deep NREM sleep by TC-specific PLCβ4 deletion. (**a**) The number of episodes of each vigilance state during the light phase. **(b)** The episodic duration of each vigilance state during the light phase. **(c)** The transition probability between wake and sleep states. **(d)** The scheme of transition probabilities among wake and sleep states in control and PLCβ4 TC KO mice. Thick (orange) and dotted (gray) arrows indicate increase and decrease of the transition tendency in PLCβ4 TC KO mice compared with control mice. The data were obtained from control (n = 10, colored bar) and PLCβ4 TC KO mice (n = 12, white bar). Data are represented as the mean ± standard error of the mean (SEM). ^*^p < 0.05; ^**^p < 0.01; ^***^p < 0.005.

To distinguish if the altered durations of light and deep NREM sleep were due to a change in either the occurrence or the maintenance of each episode, we analyzed the number and duration of episodes in each state. PLCβ4 TC KO mice showed a greater number of deep NREM episodes (control, 104.50 ± 8.41; PLCβ4 TC KO, 135.67 ± 6.15; p = 0.008, unpaired t-tests; Fig. 3a) and a significantly longer episodic duration of deep NREM (control, 80.40 ± 9.04 sec; PLCβ4 TC KO, 132.50 ± 11.62 sec; p = 0.002, unpaired t-tests; Fig. 3b). There was no significant difference in the number of light NREM episodes between groups (Fig. 3a), but PLCβ4 TC KO mice showed a significantly shorter episodic duration of light NREM (control, 129.20 ± 13.60 sec; PLCβ4 TC KO, 80.25 ± 6.56 sec; p = 0.006, unpaired t-tests; Fig. 3b). These results indicate that the total duration of deep NREM was increased by the enhancement of both occurrence and maintenance in PLCβ4 TC KO. However, the decrease in the total duration of light NREM was caused by weakened maintenance and not by occurrence.

Next, we analyzed the transition probability from one state to other states. In control mice, the transition from awake to NREM sleep was dominated by a transition from awake to light NREM (W-L.NR, ~65%, Fig. 3c), which is considered normal in human sleep^31^. However, in the PLCβ4 TC KO mice, the W-L.NR transition was reduced to ~45%, whereas W-D.NR transition increased by almost 55% (W-L.NR: control, 65.31 ± 3.26%; PLCβ4 TC KO, 45.03 ± 5.39%; p = 0.0049, unpaired t-tests; W-D.NR: control, 34.69 ± 3.26%; PLCβ4 TC KO, 54.97 ± 5.39%; p = 0.0049, unpaired t-tests; Fig. 3c). Once the light NREM sleep was initiated in PLCβ4 TC KO mice, it transitioned more frequently into deep NREM sleep than it did in control mice (L.NR-D.NR: control, 58.05 ± 3.14%; PLCβ4 TC KO, 66.42 ± 1.67%; p = 0.03, unpaired t-tests; Fig. 3c). The L.NR to REM (L.NR-R) transition was less frequent in PLCβ4 TC KO mice than control. Instead, the D.NR-R transition, which is abnormal sleep progress rarely observed in controls, was often occurred in PLCβ4 TC KO mice (L.NR-R: control, 26.63 ± 2.35%; PLCβ4 TC KO, 20.00 ± 1.16%; p = 0.02, unpaired t-tests; D.NR-R: control, 1.14 ± 0.32%; PLCβ4 TC KO, 3.40 ± 0.62%; p = 0.005, unpaired t-tests; Fig. 3c). From REM sleep, the R-L.NR transition was reduced, whereas R-D.NR transition increased in PLCβ4 TC KO mice than in control mice (R-L.NR: control, 63.74 ± 4.42%; PLCβ4 TC KO, 48.93 ± 4.76%; p = 0.03, unpaired t-tests; R-D.NR: control, 12.61 ± 2.99%; PLCβ4 TC KO, 33.73 ± 3.85%; p = 0.0003, unpaired t-tests; Fig. 3c). Altogether, these results showed that the relative transition to deep NREM sleep significantly increased in PLCβ4 TC KO mice, which resulted in a stabilized deep NREM sleep and destabilized light NREM sleep (Fig. 3d). These results indicate that changes restricted to TC neurons dramatically altered the NREM sleep composition, which is subdivided based on sleep depth. This led us to further investigate sleep-brain rhythms known to be generated in thalamocortical circuit during NREM sleep.

### **Spindle activity in light NREM sleep decreased in TC-specific PLCβ4 KO mice**

**Figure 4:**
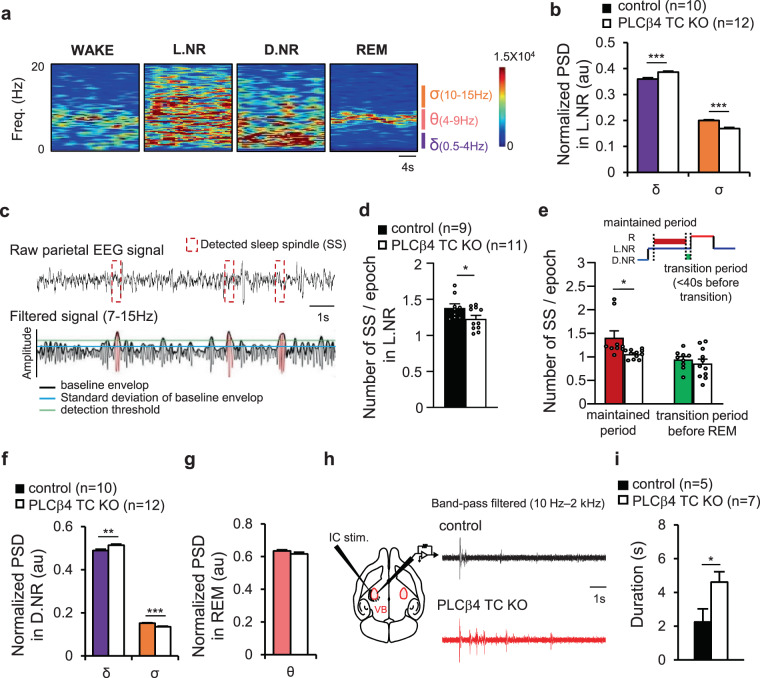
The contrast changes in brain rhythms of light and deep NREM sleep by the TC-specific PLCβ4 deletion. (**a**) Representative spectrograms in wake and sleep states. **(b)** Normalized power spectrum density (PSD) for the δ (0.5–4 Hz) and σ (10–15 Hz) bands (arbitrary units, au) in the light NREM sleep (L.NR). **(c)** Representative traces illustrating the spindle detection protocol. **(d)** The number of sleep spindle (SS) per epoch occurred in the L.NR state was significantly reduced in the PLCβ4 TC KO mice. **(e)** The number of SS per epoch occurred in the L.NR state, which was maintained (red) or right before transited to the REM sleep (green) (control, n = 9, colored bar; PLCβ4 TC KO, n = 11, white bar). **(f)** Normalized PSD for the δ and σ bands in the deep NREM sleep (D.NR). **(g)** Normalized PSD for the θ (4–9 Hz) band in REM sleep. All PSD data were obtained from control (n = 10, colored bar) and PLCβ4 TC KO mice (n = 12, white bar). **(h)** Schematic illustration of intra-thalamic oscillation recording in the ventro-posterolateral nuclei (left). Representative traces of thalamic oscillations in control and PLCβ4 TC KO mice (right). **(i)** The duration of intra-thalamic oscillatory activity increased in PLCβ4 TC KO mice (control, n = 5, black bar; PLCβ4 TC KO, n = 7, white bar). Data are represented as the mean ± standard error of the mean (SEM). ^*^p < 0.05; ^**^p < 0.01; ^***^p < 0.005.

Next, we analyzed the power of brain rhythms representing each sleep state, independent of the duration of state (Fig. 4a). In the light NREM state, the sigma (σ) band power significantly decreased with the enhancement of the δ band power in PLCβ4 TC KO mice compared with control mice (σ: control, 0.20 ± 0.003; PLCβ4 TC KO, 0.17 ± 0.004; p = 5.85 × 10^−6^, unpaired t-tests; δ: control, 0.36 ± 0.005; PLCβ4 TC KO, 0.39 ± 0.004; p = 0.0002, unpaired t-tests; Fig. 4b). Next, we counted and analyzed the spindle activity as the average number of spindles per 1 epoch (8 sec) in the light NREM state during which spindles are most commonly observed (Fig. 4c). The number of spindle per epoch decreased in PLCβ4 TC KO mice compared to control mice (control, 1.38 ± 0.05; PLCβ4 TC KO, 1.23 ± 0.05; p = 0.049, unpaired t-tests; Fig. 4d). The total number of spindles in the light NREM state also decreased in PLCβ4 TC KO mice (Supplementary Fig. S2a). In the deep NREM state, there was no significant difference in the number of spindle per epoch between groups, but the total number of spindles increased in PLCβ4 TC KO mice compared to control mice (Supplementary Fig. S2a and S2b). It has been reported that spindle occurrence increases immediately before NREM to REM transition^32^. We therefore investigated if the number of spindle events in the light NREM to REM transition period (<40 sec before transition occurrence) differed between groups, since both the number of spindle and the L.NR to R transition decreased in PLCβ4 TC KO mice (Figs. 3d and 4d). The number of spindle per epoch in the transition period was not changed in PLCβ4 TC KO mice compared to control mice. However, the spindle occurring in the maintained light NREM period, excluding the transition period, significantly decreased in PLCβ4 TC KO mice (control, 1.40 ± 0.15; PLCβ4 TC KO, 1.06 ± 0.03; p = 0.047, unpaired t-tests; Fig. 4e). These results indicate that TC-specific PLCβ4 KO decreased the spindle occurrence in the maintained light NREM period.

In the deep NREM state, δ band power significantly increased, with the reduction of the σ band power in PLCβ4 TC KO mice compared with control mice (δ: control, 0.49 ± 0.005; PLCβ4 TC KO, 0.51 ± 0.005; p = 0.006, unpaired t-tests; σ: control, 0.15 ± 0.002; PLCβ4 TC KO, 0.14 ± 0.003; p = 9.1 × 10^−5^, unpaired t-tests; Fig. 4f). In the REM state, there was no change in θ band power between groups (Fig. 4g). The slow intra-thalamic oscillatory activity^33^, which was recorded in horizontal thalamocortical slices *in vitro*, was enhanced in PLCβ4 TC KO mice (control, 2.26 ± 0.77 sec; PLCβ4 TC KO, 4.61 ± 0.61 sec; p = 0.042, unpaired t-tests; Fig. 4h,i), which was parallel with increased δ power in NREM sleep. These results indicate that spindle activity and δ band power were regulated via a distinct mechanism, although both brain rhythms were generated in thalamocortical circuit.

### **Declarative memory consolidation was impaired in TC-specific PLCβ4 KO mice, regardless of longer NREM sleep**

**Figure 5:**
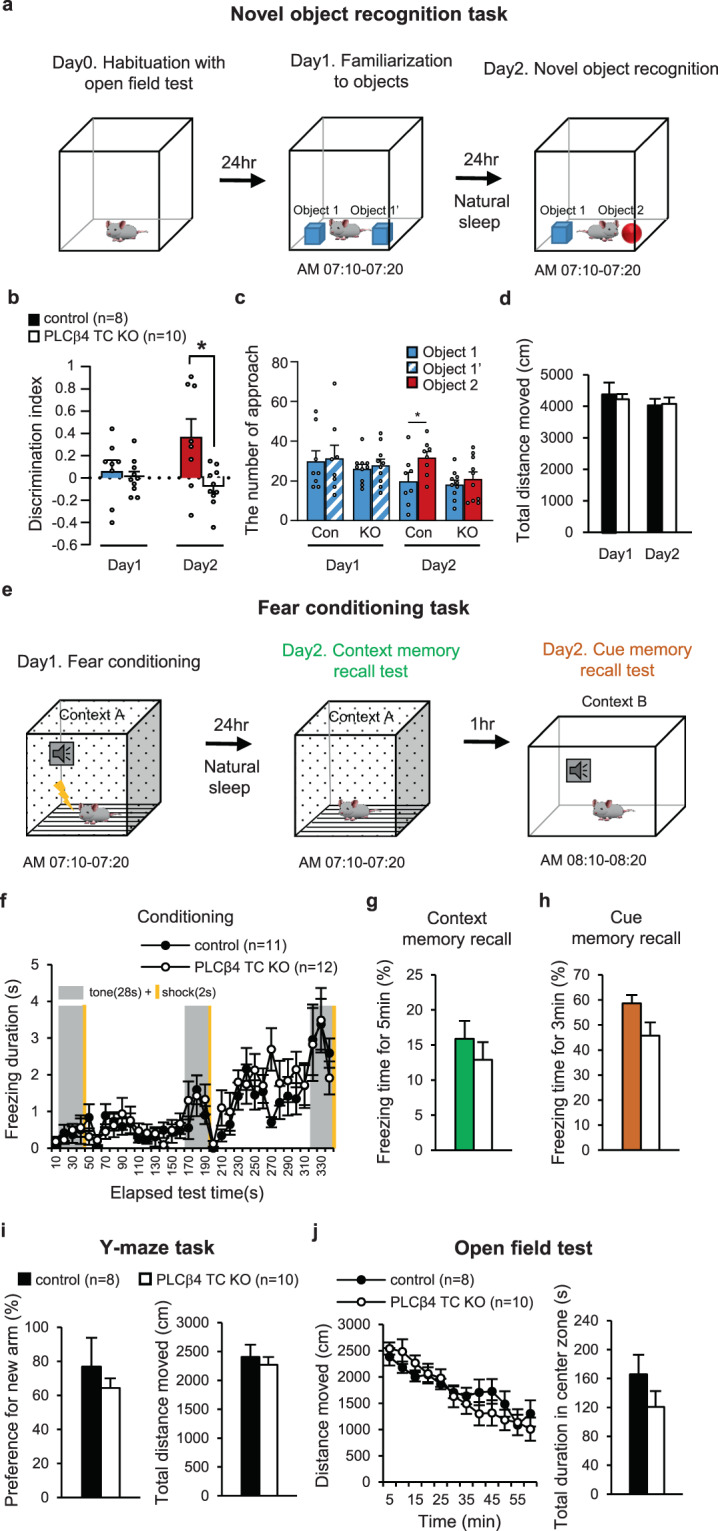
Sleep-dependent declarative memory impairment by TC-specific PLCβ4 deletion. (**a**) A schematic illustration of the novel object recognition task. **(b)** The discrimination index during the training (day 1) and test session (day 2) (control, n = 8, colored bar; PLCβ4 TC KO, n = 10, white bar). **(c)** The number of approach for each object during the training and test session (control, n = 8; PLCβ4 TC KO, n = 10; blue bar, object 1; blue grid bar, object 1’, red bar, object 2). **(d)** The total distance moved for 10 min during the training and test session (control, n = 8, black bar; PLCβ4 TC KO, n = 10, white bar). **(e)** A schematic illustration of the fear conditioning task. **(f)** Time course of the freezing behavior during the conditioning phase. The conditioning session consisted of a 28-sec tone (gray box) followed by a 2-sec shock (yellow line) delivered at 2-min intervals. Each point represents a 10-sec bin. **(g,h)** Freezing time during the context- and cue-memory recall tests. (control, n = 11, colored bar; PLCβ4 TC KO, n = 12, white bar). **(i)** Y-maze task for testing the sleep-independent memory function. (control, n = 8, black bar; PLCβ4 TC KO, n = 10, white bar). **(j)** Open field test for testing locomotor activity (left) with the anxiety level (right) (control, n = 8, black bar; PLCβ4 TC KO, n = 10, white bar). Data are represented as the mean ± standard error of the mean (SEM). ^*^p < 0.05.

Longer NREM sleep is reported to benefit memory formation^14^. We investigated the effect of increased NREM sleep duration in TC-specific PLCβ4 KO mice on sleep-dependent memory consolidation. First, a novel object recognition task was performed to test declarative memory (Fig. 5a). Strikingly, the discrimination index (DI) was significantly lower in PLCβ4 TC KO mice than in control mice (day 2: control, 0.37 ± 0.16; PLCβ4 TC KO, −0.08 ± 0.06; p = 0.028, unpaired t-tests; Fig. 5b), which indicates that memory consolidation was impaired. During the task, the difference between two groups was not observed in the approach number to objects (except for new object) or total distance moved (Fig. 5c,d). These showed that PLCβ4 TC KO mice has normal sensory and cognitive functions like the control mice. To examine non-declarative memory function, the same mice completed a fear conditioning task and both control and PLCβ4 TC KO mice showed normal learning function in the conditioning session (Fig. 5e,f). There were no between-group differences in the context- and cue-recall memory consolidations (Fig. 5g,h).

Next, sleep-independent memory function was tested using a Y-maze task, in which sleep was not permitted between the training and test sessions. There was no change in the preference for a novel arm between groups, which indicated that sleep-independent memory consolidation was normal in PLCβ4 TC KO mice (Fig. 5i). Both groups showed general locomotor activities with normal anxiety levels in the open field test (Fig. 5j). These results indicate that the TC-specific PLCβ4 deletion impaired sleep-dependent declarative memory consolidation, despite the increase in NREM sleep duration. These results prompted us to further investigate which detailed sleep components, such as stability of each episode, transition pattern between vigilance states and brain rhythms, rather than just the total NREM sleep duration, were related to memory deficits in PLCβ4 TC KO mice.

### **Memory consolidation was positively correlated with light NREM stability**

**Figure 6:**
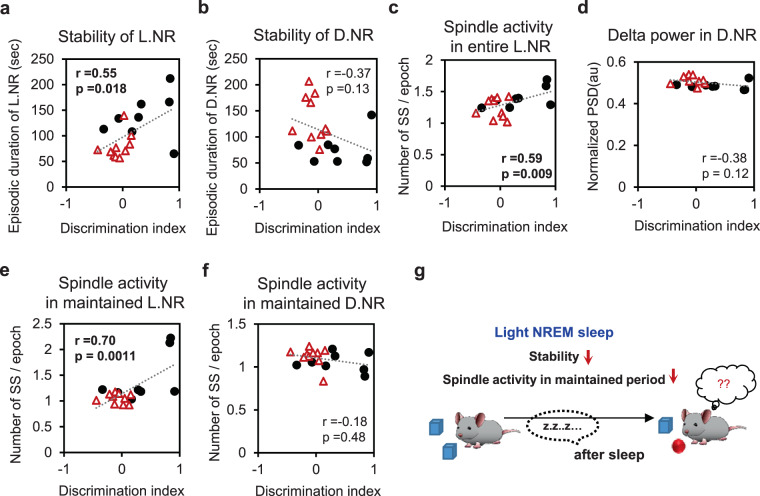
Differential correlation of light and deep NREM sleep components with memory consolidation. (**a**) The correlation between the episodic duration of the light NREM sleep and the discrimination index (DI) for a novel object. **(b)** The correlation between the episodic duration of the deep NREM sleep and the DI for novel object. **(c)** The correlation between the number of sleep spindle (SS) per epoch occurring in the entire light NREM sleep and the DI for novel object. **(d)** The correlation between the normalized δ power in the deep NREM sleep and the DI for novel object. **(e)** The correlation between the number of SS per epoch occurring in the maintained light NREM period and the DI for novel object. **(f)** The correlation between the number of SS per epoch occurring in the maintained deep NREM period and the DI for novel object. Each point represents a value obtained from one mouse (n = 18; control, black circle; PLCβ4 TC KO, red triangle). **(g)** A scheme summarizing the importance of light NREM sleep for declarative memory consolidation via its stability and spindle activities.

TC-specific PLCβ4 KO mice were characterized by a destabilized light NREM sleep, but with a stabilized deep NREM sleep (Fig. 3d). We therefore performed regression analyses between the degree of memory consolidation and components in the light and deep NREM sleep in all the mice tested. The degree of memory consolidation was expressed as a DI for a novel object. Among the many components of light NREM sleep, the episodic duration, indicating stability, showed a significant positive correlation with memory consolidation (r = 0.55; p = 0.018; Fig. 6a). However, there was no correlation between the episodic duration of deep NREM sleep and memory consolidation. (r = −0.37; p = 0.13; Fig. 6b). Interestingly, the total amount of NREM for 12 hr, especially deep NREM sleep, had negative correlation with memory consolidation (NR: r = −0.48; p = 0.04; D.NR: r = −0.48; p = 0.04; Supplementary Fig. S3a and S3b). These results suggest that light NREM sleep tightly linked to the consolidation of declarative memory via its stability, irrespective of the total amount of NREM sleep (Fig. 6g).

We then determined whether the decreased spindle activity in light NREM (Fig. 4d) or enhanced δ power in deep NREM (Fig. 4f) contributed more to the memory deficit in PLCβ4 TC KO mice. There was a strong positive correlation between the number of spindle per epoch occurring in the light NREM sleep and memory consolidation (r = 0.59; p = 0.009; Fig. 6c). However, δ power of the deep NREM sleep did not correlate with memory consolidation (r = −0.38; p = 0.12, Fig. 6d). Overall, these results indicate that spindle activity during light NREM sleep has a positive effect on declarative memory consolidation (Fig. 6g).

Sleep spindles occur throughout NREM sleep and are not limited to light NREM sleep. However, it is not known whether the role of spindles in memory functions is temporal-dependent. To investigate this, we divided the spindle into those occurring in maintained light or deep NREM periods and those occurring in the transition period from light to deep NREM sleep. A strong correlation with memory consolidation was shown only when the spindle occurred in maintained light NREM period (r = 0.70; p = 0.0011; Fig. 6e). There was no correlation between the number of spindle per epoch in maintained deep NREM period and memory consolidation (r = −0.18; p = 0.48; Fig. 6f). Furthermore, the number of spindle per epoch in the transition period from light to deep NREM did not correlate with memory consolidation (r = −0.04; p = 0.88; Supplementary Fig. S3c). These results indicate that the positive effect of spindle activity on declarative memory consolidation was specific for spindles that occurred during the maintained light NREM period (Fig. 6g).

## Discussion

Both NREM and REM sleep have been reported to positively contribute to memory function^34^. However, most studies have highlighted the importance of deep NREM or REM sleep in memory formation, and the role of light NREM sleep dynamics is not clear^17^. In the present study using TC-specific PLCβ4 KO mice, we demonstrate that opposite changes of composition in light and deep NREM sleep and their effect on memory consolidation.

NREM sleep accompanied large-amplitude oscillatory EEG patterns, which presumably reflect the synchronized oscillations in thalamocortical circuit, although the initiation of NREM has been mostly attributed to the preoptic area of the hypothalamus^35^. Both sleep spindles and δ waves, which predominantly occurred in light and deep NREM sleep, respectively, are known to be generated in thalamocortical circuit accompanying burst firing in TC neurons^19,20^. We previously reported that deletion of PLCβ4 dramatically increased burst firing in TC neurons^26^. We therefore investigated whether TC-specific PLCβ4 KO would affect NREM sleep dynamics and NREM sleep rhythms. TC-specific PLCβ4 KO increased the total duration of NREM sleep, but not REM sleep compared to whole PLCβ4 KO which showed the changes in many aspects of sleep components probably due to the expression of PLCβ4 in other brain regions including brain stem and medial septum^36^. Especially, PLCβ4 TC KO mice displayed a significant increase in the duration of deep NREM sleep that occurred via enhancement of its occurrence and stability, which led to an increase in the total sleep duration. However, the duration of the light NREM sleep was reduced, with a weakened stability and no change in its occurrence (Fig. 3d). In brain rhythms, the spindle incidence in the light NREM state was reduced (Fig. 4d), although burst firing increased in PLCβ4 TC KO mice^26^. This result supports previous reports that suggest that TC burst firing is not essential for spindle generation^33^. Interestingly, the change of spindle incidence per epoch by PLCβ4 TC KO was observed only in light NREM state, but not deep NREM state (Fig. 4d and Supplementary Fig. S2b). This supports a previous proposal that fast spindles, which are most pronounced in light NREM sleep, are generated in thalamic nuclei, whereas slow spindles, which are most evident in deep NREM sleep, originate from the intracortical area, as well as thalamic region^37,38^.

Many studies have suggested that an increase in NREM sleep duration facilitates declarative memory processing^39^. However, we found that sleep-dependent declarative memory consolidation was impaired, despite the increase in NREM sleep duration (Fig. 5b). We observed a strong and positive correlation between declarative memory consolidation and the episodic duration of light NREM sleep in all tested animals together (Fig. 6a). When separating the control and PLCβ4 TC KO groups, their positive correlation was observed only in the control group, suggesting that the stability of light NREM and memory consolidation has correlational nature. The correlation disappeared in PLCβ4 TC KO group where the light NREM stability was decreased (Supplementary Fig. S4a). A previous human study reported that the duration of light NREM sleep was positively correlated with procedural memory function^40^. Our results indicate that the stability of light NREM sleep has beneficial role in declarative memory functions. On the other hand, we found a negative correlation between total amount of deep NREM sleep and declarative memory consolidation, which conflicts with the results of several human studies that have reported deep NREM sleep to enhance the memory formation^16,41^. However, most of these previous studies could not exclude the effect involving enhancement of light NREM sleep for memory improvement because of limitations in their experimental method. Therefore, our results suggest that the abnormal increase in deep NREM sleep accompanied by a loss of light NREM sleep may impair the memory formation.

There have been diverse opinions about the role of δ waves, which are nested within slow sleep oscillations, in memory formation. The δ waves were considered to promote the performance of various types of memory^42^. However, in patients with schizophrenia, δ power during deep NREM sleep is not associated with declarative memory function^43^. In the present study, we did not observe any correlation between the δ power in deep NREM state and declarative memory consolidation, regardless of PLCβ4 deletion (Fig. 6d, Supplementary Fig. S4e).

Our data demonstrated that the spindle activity in the light NREM state had a positive correlation with declarative memory consolidation in all tested animals together (Fig. 6c). Their positive correlation was conserved in the control group, suggesting the correlational nature between the spindle activity of light NREM state and memory consolidation (Supplementary Fig. S4b). This supports that the decreased spindle activity of light NREM state would be linked to the sleep-dependent memory impairment observed in PLCβ4 TC KO mice. Despite the fact that spindles are observed throughout NREM sleep, many researchers, suggesting the importance of spindles for memory function, have highlighted light NREM sleep, where the spindles frequently occur^44^. It is unknown whether spindle activity that occurs during light vs. deep NREM or the maintained vs. transition period of NREM has a distinct role in memory function. We found a strong correlation with memory consolidation was observed when spindle occurred during the maintained light NREM period only and not during deep NREM or the transition period (Fig. 6e,f; Supplementary Fig. S3c). These results were confirmed when the control group was analyzed separately (Supplementary Fig. S4c and S4f). These results indicate that the positive role of spindles on memory formation depends on when the spindle occurs.

In summary, we demonstrated that declarative memory consolidation was positively correlated with the stability of light NREM sleep, irrespective of the total amount of NREM sleep. Moreover, this is the first study to report that the timing of spindle occurrence in light NREM state may be critical for the beneficial effect of spindles on memory consolidation. These findings highlight the importance of light NREM sleep for memory formation, and provide novel insights into a balance between light and deep NREM sleep, not simple sleep induction, as therapeutic and diagnostic targets for the treatment of sleep-related cognitive deficits.

## Methods

### Study animals

All experiments used *Plcβ4* floxed mice from a C57BL/6J genetic background. The mice were maintained with free access to food and water under a 12-h light and 12-h dark cycle, with the light cycle beginning at 7:00 a.m. Animal care and handling were conducted in accordance with the guidelines of the Institutional Animal Care and Use Committee (IACUC) at Yonsei University (Seoul, Korea) and all experimental protocols were apporoved by the IACUC at Yonsei University (Approval ID number IACUC-A-201601-407-02).

### Surgery

Twelve- to 14-week-old male mice were used for the chronic monitoring of the EEG/EMG signals. The mice were anesthetized with 0.2% tribromoethanol (20 ml/kg, intraperitoneal injection; Sigma-Aldrich, St. Louis, MO, USA) and placed on a stereotaxic frame (David Kopf Instruments, Tujunga, CA, USA). Cre-inducible vectors containing adeno-associated virus (AAV9.hsyn.HI.eGFP-Cre.WPRE.SV40) were bilaterally injected into the ventrobasal complex region of the thalamus (anteroposterior, −1.82 mm; lateral, ±1.75 mm; ventral, −3.5 and −3.0 mm, 1 μℓ in each site) using a NanoFil needle (NF33BL; World Precision Instruments, Sarasota, FL, USA) with a Hamilton syringe and pump (SP100IZ; World Precision Instruments, USA). AAV9.hsyn.eGFP.WPRE.bGH was used as control. For the EEG recordings, epidural electrodes with a 6-pin surface mount connector with EMG leads (8231-SM; Pinnacle Technology, USA) were implanted in the frontal and parietal lobes. For the EMG signal recordings, EMG leads were inserted into the nuchal musculature and a grounding electrode was implanted in the occipital region of the skull.

### Chronic EEG/EMG monitoring

After a 2-week recovery, mice were placed in unrestrained chronic recording environments under 12-h light and 12-h dark conditions. There was a 1-week adaptation period to the recording systems. EEG and EMG signals were chronically acquired for 24 h using a three-channel biopotential recording system and Sirenia software (Pinnacle Technology, USA, RRID:SCR_016183). The signals were collected using a preamplifier (8202 mouse preamplifier for a 3-channel system; Pinnacle Technology, USA) with a low-pass filter at 400 Hz for EEG and high-pass filter at 10 Hz for EMG, and digitized at a sampling rate of 400 Hz (8206, EEG/EMG data conditioning and acquisition system; Pinnacle Technology, USA).

### Novel object recognition procedures and behavioral analyses

On day 0, an open field test was performed for 60 min in a quadratic recording box (40 × 40 × 40 cm) made of acrylic white polyvinyl chloride, which was the habituation session for the novel object recognition task; continuous video tracking was implemented to identify and exclude mice with abnormal locomotion. On the following day, the test was run on 2 consecutive days during the first 2 h of the light cycle onset (7:00 am to 9:00 am). On day 1, the mice were placed in a quadratic recording box and the same two identical objects (object 1) were each placed in a randomly assigned quadrant within the test area. The mice were allowed to explore freely for 10 min before being returned to their sleep recording chamber. On day 2, one of the objects was randomly replaced by a new one (object 2). The mice were given 10 min to explore the test area. Test sessions on each day were recorded with an overhead video camera and analyzed using Ethovision XT (Noldus, Leesburg, VA, USA, RRID: SCR_000441). For the analysis of novel object preferences, the time spent exploring each object during the test period was measured for each mouse and preference was determined using the following equation: Discrimination index (DI) = (object 2 exploration (sec) − object 1 exploration (sec)/(object 1 exploration (sec) + object 2 exploration (sec)). On day 1, the novel object randomly indicated one of two objects and mice with side preference was excluded in data set (control, n = 2; PLCβ4 TC KO, n = 2).

After the experiment, all mice were placed in a Y-maze composed of three equally spaced arms (120°; 30 cm long). The arms were labeled by different patterns of masking tape to distinguish each arm and defined as A, B, and C. In the training session, mice were allowed to move freely in two compartments (A and B) for 5 min before being returned to their home cage. After a 30-min break, the mice were again given 5 min to explore the test area, in which all three compartments (A, B, and C) were open. Short-term memory was indicated by the preference for a new arm and was determined using the following equation: new arm preference (%) = exploration time in C (sec)/exploration time in A + B (sec) × 100.

### Fear conditioning procedures and behavioral analyses

All mice were tested for context- and cue-based responses following a standard fear conditioning protocol. The tests were implemented within the first 2 h of the onset of the light cycle (7:00 a.m. to 9:00 a.m.). During the conditioning phase, mice were placed in a metallic rectangular chamber with a surface grid (context A) connected to an electrical shocker (Coulbourn Instruments, Hollistan, MA, USA). The mice were habituated to the box for 4 min, followed by application of three tone-shock pairs separated by an interval of 2 min. Each tone (2 kHz at 80 dB) lasted for 28 sec and was accompanied by a foot shock (2 sec, 0.5 mA). Once conditioning was completed, the mice were returned to their sleep recording chamber. Between each conditioning session, the test area was cleaned with 70% ethanol and water.

On the following day, contextual and cued memory recall tests were completed 1 h apart within the first 2 h of the onset of the light cycle. For the context test, the mice were placed in the same chamber, as they had been for context A during conditioning, for 5 min; however, no tone-shock events were delivered. Contextual fear memory was determined by the percentage of freezing time for 5 min. Upon test completion, the mice were immediately returned to their sleep recording chamber for ~1 h. The test area was thoroughly cleaned with 70% ethanol and water between subsequent test sessions.

For cue testing, the test area (context B) was substantially different than that used for the conditioning session and context testing. The floor and walls were white Plexiglass panels, as opposed to the black walls of the testing area used for conditioning and context tests. Peroxyguard was used to clean the test area between mice. During the cue test, three 80 dB tones of 28 sec separated by an interval of 2 min, identical to those produced during conditioning, were presented. Cue fear to the tone was determined by the percentage of freezing time during a 3-min period (3 tone × 1 min following tone onset). For both the contextual and cue fear tests, freezing behavior was assessed by a blinded experimenter using behavior counter software, and freezing durations in the conditioning phase were presented across 10-sec bins.

### Sleep scoring and analysis

The parietal EEG/EMG recordings were scored semi-automatically using the SleepSign software sleep scoring system (Kissei Comtec, USA) in 8-sec epochs as awake (low voltage, high frequency EEG activity with a high amplitude EMG), NREM sleep (high amplitude, slow EEG activity with a reduction of the EMG tone), and REM sleep (low amplitude EEG comprised mainly of θ-wave activity with EMG atonia). NREM sleep was further subdivided into the light and deep NREM by the ratio of the δ frequency (0.5–4 Hz) power^27–30^. In each epoch, the total EEG power within the 0–20 Hz frequency range was normalized to 100%. When the percentage of δ frequency power to total EEG power was less than 40% in one epoch, it was scored to the light NREM sleep. Deep NREM sleep was scored when the percentage of δ frequency power to total EEG power was higher than 40% in one epoch. The onset of sleep and wake episodes was defined as three consecutive epochs. Epochs containing artifacts that occurred during active wakefulness (with large movements) or containing two vigilance states were visually identified. Transition probability (from A to B state) was determined using the following equation: transition number to B/total number of transitions from A × 100.

### Power spectral density analysis

To analyze the power spectral densities, the EEG spectral power in each 8-sec epoch was calculated in 0.39-Hz bins using fast Fourier transformation (Hamming window) and normalized within each sleep state using SleepSign software. The power bins in the 0–20 Hz range were summed for the three frequency bands [δ (0.5–4 Hz), θ (4–9 Hz), σ (10–15 Hz)] and then averaged within each group.

### The spindle detection method

The incidence of sleep spindles during NREM sleep was analyzed using MATLAB software (MathWorks, RRID: SCR_001622). EEG traces of the light NREM state were obtained by dividing into period to be maintained or before transition to deep NREM or REM sleep. EEG traces of the deep NREM state were obtained in maintained periods. The transition period was defined as the five epochs before transition to other states or immediately after the transition. The epochs, except for those comprising the transition period, were selected as maintained periods. The EEG signal was bandpass filtered at 7–15 Hz and the envelope was determined using the Hilbert transform. A potential spindle event was identified in cases where the envelope exceeded a threshold, which was defined as the standard deviation of the baseline envelope multiplied by 2. The spindles by identifying the center of detected spindles was presented as the number of spindle occurring in one epoch (8 s) and averaged first for each mouse and then within groups. Subsequently, potential spindle events with durations shorter than 0.2 sec or longer than 3 sec were discarded.

### Immunohistochemistry

For histological analysis, mice were anesthetized with 0.2% tribromoethanol (20 ml/kg, intraperitoneal injection) and transcardially perfused with 1 M phosphate-buffered saline (PBS) followed by a 4% paraformaldehyde solution. After perfusions, the brains were fixed in 4% paraformaldehyde overnight and then submerged in 30% sucrose solution for 3 days at 4 °C. The brains frozen in O.C.T. compound were cut into serial 40-μm thick coronal sections on a freezing microtome, and stored in PBS. The brain sections were permeabilized with 0.1% Tween-20 in PBS for 30 min and then incubated in blocking solution (5% normal goat serum in PBS) for 1 h. After washing three times with PBS, the tissues were incubated with a primary antibody against PLCβ4 (H-300; Santa Cruz Biotechnology, Santa Cruz, CA, USA; 1:200) for 24 h at 4 °C. The tissues were rinsed three times in PBS, incubated with a Cy3-conjugated secondary antibody (Jackson ImmunoResearch Labs, West Grove, PA, USA, RRID: AB_2338000; 1:500) for 2 h at room temperature, and then mounted on microscope slides with fluorescent mounting media (Dako, Glostrup, Denmark). Fluorescence images were obtained with a LSM 880 confocal microscope (Carl Zeiss, Oberkochen, Germany). The image was acquired in consistent parameters across the conditions and selected the middle of the TC region by the experimenter blinded to the conditions. The cell counting was performed manually from the two other blinded experimenters.

### Quantitative reverse transcription-PCR (qRT-PCR)

Total RNA was isolated from the thalamus of *Plcβ4* floxed mice injected with AAV.eGFP or AAV.eGFP-Cre, using TRIzol reagent (Invitrogen, Carlsbad, CA, USA). One µg of total RNA was used for cDNA synthesis with the SuperScript III First-Strand Synthesis System for RT-PCR (Invitrogen, Carlsbad, CA, USA). qRT-PCR was performed using the CFX Connect Real-Time PCR Detection System (Bio-Rad, Hercules, CA, USA). The qRT-PCR primers used to assess expression were as follows: *β-actin*: F1 primer 5′-TGTGATGGTGGGAATGGGTCAGAA-3′ and R1 primer 5′-TGTGGTGCCAGATCTTCTCCATGT-3′ to produce a 140 bp cDNA; *Plcβ1*: F2 primer 5′-TCGTACATCCAGGAGGTGGT-3′ and R2 primer 5′-CTGCAGCTTGGGCTTCTCAT-3′ to produce a 129 bp cDNA; *Plcβ4* primer 1: F3 primer 5′-CTGGAAGGGCGGATATTGTGT-3′ and R3 primer 5′-ATCGGACTGACGTTGTTTGC-3′ to produce a 155 bp cDNA; *Plcβ4* primer 2: F4 primer 5′-GGGCGGATATTGTGTGTCTG-3′ and R4 primer 5′-TGTTGGTCAGAAAGGCCAGTT-3′ to produce a 196 bp cDNA. Gene expression was quantified using the comparative CT method with *β-actin* as the reference gene.

### *In vitro* intra-thalamic oscillation recordings

For intra-thalamic oscillation recordings, mice were anesthetized by halothane and decapitated. The brains were sectioned to achieve 400 µm-thick horizontal slices using VT1000s (Leica Microsystems, Wetzlar, Germany). The slices were stabilized for at least 1 h in artificial cerebrospinal fluid (aCSF) containing NaCl, 130 mM; NaHCO_3_, 24 mM; KCl, 3.5 mM; NaH_2_PO_4_, 1.25 mM; CaCl_2_, 1.5 mM; MgCl_2_, 0.75 mM; and glucose, 10 mM, and simultaneously equilibrated with 95% O_2_/5% CO_2_ at 25 °C. After stabilization, intra-thalamic oscillations were evoked by a 300 µA and 100 µs square pulse stimulus in the internal capsule (IC) through a bipolar tungsten electrode (FHC, Bowdoin, ME, USA). The stimulus interval was 30 sec. Extracellular potentials evoked by IC stimulation were measured in ventro-posterolateral nuclei of thalamus using a tungsten electrode (~100 kΩ). Signals were amplified using MultiClamp 700B (Molecular Devices, San Jose, CA, USA, RRID:SCR_011323), and data acquisition was performed using a Digitizer 1440 A and Clampex (Molecular Devices). The measured signals were bandpass filtered at 10 Hz–2 kHz.

### *In vitro* whole-cell patch-clamp recordings

The burst firings were recorded under aCSF solution (NaCl, 124 mM; KCl, 3 mM; MgSO_4_, 1.3 mM; NaH_2_PO_4_, 1.25 mM; NaHCO_3_, 26 mM; CaCl_2_-2H_2_O, 2.4 mM; and glucose, 10 mM) and aerated with 95% O_2_/5% CO_2_ by whole-cell current-clamp. Patch electrodes (4-7MΩ) fabricated from standard-wall borosilicate glass (GC150F-10, Warner Instrument Corp., USA) were filled with an intra-pipette solution containing K-gluconate, 145 mM; HEPES, 10 mM; NaCl, 5 mM; EGTA, 0.2 mM; Mg-ATP, 5 mM; and Na_2_-GTP, 0.5 mM, with pH adjusted to 7.3 and osmolality adjusted to 285~295 mOsmol/kg. Rebound T-type calcium channel-mediated burst firing was measured after one-second-hyperpolarizing current step. The number of intra-burst spike was identified in cases where the amplitude of action potential exceeded a threshold, which was defined as “dV/dt (mV/ms) > 10 mV/ms”^45,46^.

### Statistical analysis

All data were initially confirmed that is well-modeled by a normal using D’Agostino-Pearson normality test. Between-group differences were analyzed using unpaired *t*-tests with Welch’s correction. Data are presented as the mean ± standard error of the mean (SEM) and a value of p < 0.05 was considered statistically significant. Linear correlation was assessed by simple linear regression analysis using GraphPad Prism 8.3.1 (GraphPad Software, San Diego, CA, USA).

## Supplementary information

Supplemantary information

## Data Availability

The datasets generated during and/or analyzed during the current study are available from the corresponding author on reasonable request.
