# Supplementary material for: Destabilization of light NREM sleep by thalamic PLCβ4 deletion impairs sleep-dependent memory consolidation
Source: Sci Rep. 2020 Jun 1;10:8813. doi: 10.1038/s41598-020-64377-7 (PMC7264240; doi:10.1038/s41598-020-64377-7)
Supplement: Supplementary file 1 — Supplemantary information [file 41598_2020_64377_MOESM1_ESM.pdf]

Supplementary Information

Title: Destabilization of light NREM sleep by thalamic PLCβ4 deletion impairs sleep-dependent memory consolidation

Authors: Joohyeon Hong, Go Eun Ha, Hankyul Kwak, Yelin Lee, Hyeonyeong Jeong, Pann-Ghill Suh, Eunji Cheong*

Supplementary Figure S1.

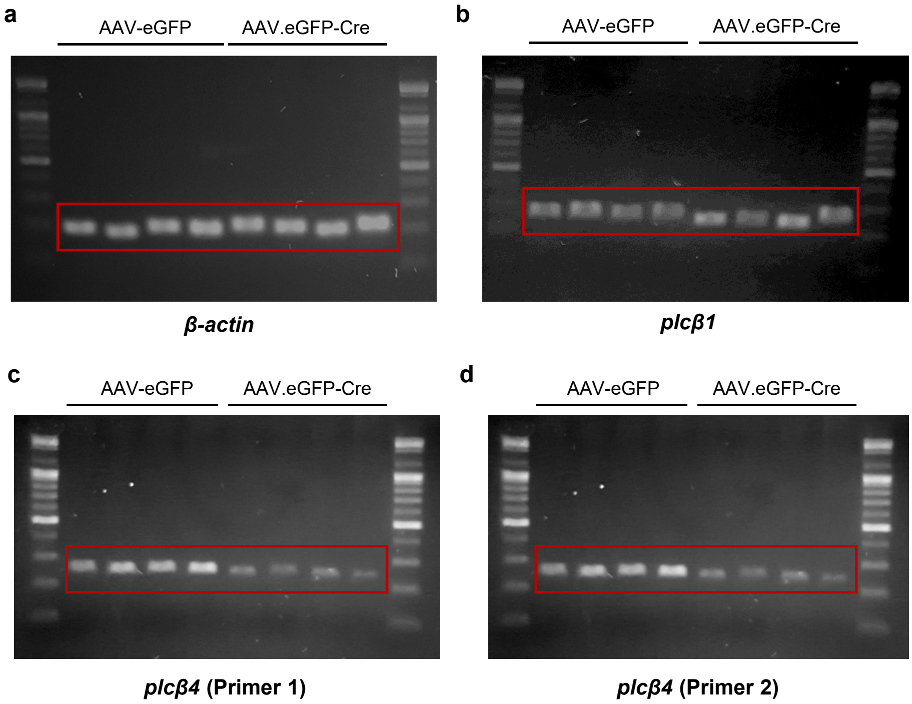

Supplementary Figure S1. Uncropped RT-PCR images from main figure. (a) Gel from Figure 1b for *β-actin*. **(b)** Gel from Figure 1b for *plcβ1*. **(c)** Gel from Figure 1b for *plcβ4* (Primer1). **(d)** Gel from Figure 1b for *plcβ4* (Primer2).

Supplementary Figure S2.

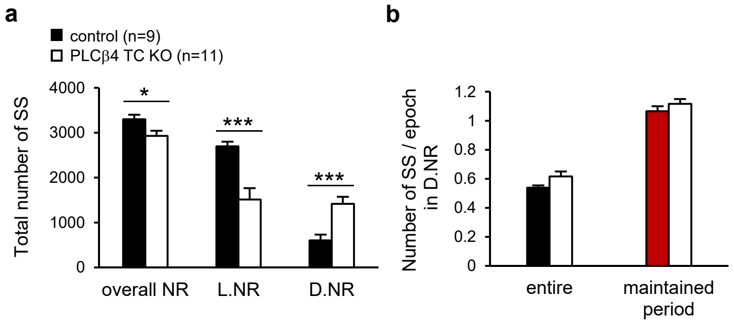

Supplementary Figure S2. Spindle incidence in the light and deep NREM sleep. (a) Total number of sleep spindle (SS) occurred in the overall, light (L.NR) and deep (D.NR) NREM sleep during the light phase. **(b)** The number of SS per epoch in entire deep NREM and maintained deep NREM period. Data are represented as the mean ± SEM (control, n = 9, colored bar; PLCβ4 TC KO, n = 11, white bar). *$p < 0.05$; ***$p < 0.005$.

Supplementary Figure S3.

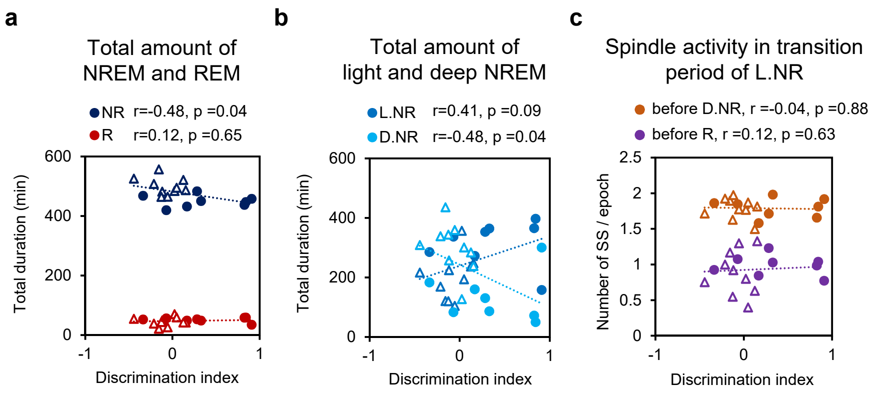

Supplementary Figure S3. Correlation analysis between sleep components and memory consolidation. (a) The correlation between the total duration of NREM (NR) or REM (R) sleep and the discrimination index (DI) for novel object. **(b)** The correlation between the total duration of light (L.NR) or deep (D.NR) NREM sleep and the DI for novel object. **(c)** The correlation between the number of sleep spindle (SS) per epoch occurring in the transition period of L.NR and the DI for novel object. Each point represents a value obtained from one mouse (n = 18; control, colored circle; PLCβ4 TC KO, open triangle).

Supplementary Figure S4.

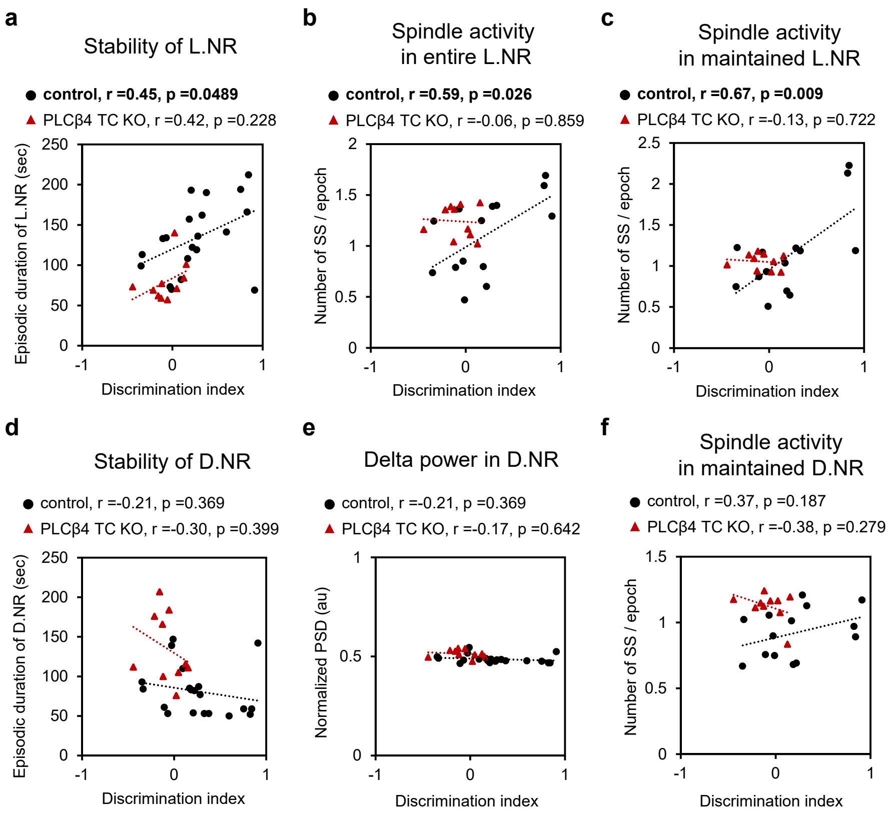

Supplementary Figure S4. Correlation analysis between sleep components and memory consolidation in separated control and PLCβ4 TC KO group. (a) The correlation between the episodic duration of the light NREM sleep and the discrimination index (DI) for a novel object (control, n = 20, black circle; PLCβ4 TC KO, n=10, red triangle). **(b)** The correlation between the number of sleep spindle (SS) per epoch occurring in the entire light NREM sleep and the DI for novel object (control, n = 14, black circle; PLCβ4 TC KO, n=10, red triangle). **(c)** The correlation between the number of SS per epoch occurring in the maintained light NREM period and the DI for novel object (control, n = 14, black circle; PLCβ4 TC KO, n=10, red triangle). **(d)** The correlation between the episodic duration of the deep NREM sleep and the discrimination index (DI) for a novel object (control, n = 20, black circle; PLCβ4 TC KO, n=10, red triangle). **(e)** The correlation between the normalized δ power in the deep NREM sleep and the DI for novel object (control, n = 20, black circle; PLCβ4 TC KO, n=10, red triangle). **(f)** The correlation between the number of SS per epoch occurring in the maintained deep NREM period and the DI for novel object (control, n = 14, black circle; PLCβ4 TC KO, n=10, red triangle). Each point represents a value obtained from one mouse.
